# COVID-19 and psychological distress: Lessons for India

**DOI:** 10.1371/journal.pone.0255683

**Published:** 2021-08-04

**Authors:** Vaijayanthee Anand, Luv Verma, Aekta Aggarwal, Priyadarshini Nanjundappa, Himanshu Rai

**Affiliations:** 1 Humanities and Social Sciences, Indian Institute of Management, Indore, Madhya Pradesh, India; 2 School of Aeronautics, Neemrana, Rajasthan, India; 3 Operations Management and Quantitative Techniques, Indian Institute of Management, Indore, Madhya Pradesh, India; 4 Department of Management Studies, Indian Institute Of Technology, Chennai, Tamil Nadu, India; 5 Human Resources and Organizational Behaviour, Indian Institute of Management, Indore, Madhya Pradesh, India; Imam Abdulrahman Bin Faisal University, SAUDI ARABIA

## Abstract

**Purpose:**

The COVID-19 pandemic has undoubtedly altered the routine of life and caused unanticipated changes resulting in severe psychological responses and mental health crisis. The study aimed to identify psycho-social factors that predicted distress among Indian population during the spread of novel Coronavirus.

**Method:**

An online survey was conducted to assess the predictors of distress. A global logistic regression model was built, by identifying significant factors from individual logistic regression models built on various groups of independent variables. The prediction capability of the model was compared with the random forest classifier.

**Results:**

The respondents (*N* = 1060) who are more likely to be distressed, are in the age group of 21-35 years, are females (OR = 1.425), those working on site (OR = 1.592), have pre-existing medical conditions (OR = 1.682), do not have health insurance policy covering COVID-19 (OR = 1.884), have perceived seriousness of COVID-19 (OR = 1.239), have lack of trust in government (OR = 1.246) and whose basic needs’ fulfillment are unsatisfactory (OR = 1.592). The ones who are less likely to be distressed, have higher social support and psychological capital. Random forest classifier correctly classified 2.3% and 17.1% of people under lower and higher distress respectively, with respect to logistic regression.

**Conclusions:**

This study confirms the prevalence of high distress experienced by Indians at the time of COVID-19 and provides pragmatic implications for psychological health at macro and micro levels during an epidemiological crisis.

## 1 Introduction

The COVID-19 pandemic has been affecting the world at an alarming rate, leaving it in shambles. According to the World Health Organization(WHO), in the month of March 2020, over 14, 000 people died due to the novel Coronavirus, with more than 3, 34, 000 being infected [1]. In India, 1, 251 confirmed COVID cases and 32 death cases were reported when nationwide lockdown was declared on 23rd March, 2020. The cases have risen sharply since then, and currently more than 10 million confirmed cases and nearing 1, 56000 death cases have been reported till date.

The spread of the disease was followed with a series of lockdown and stringent quarantine measures in various parts of the world, such as the travel restrictions, closure of educational institutions, offices, and commercial activities in India. Strict quarantine measures were prescribed to reduce the rate of infections in the country. India currently stands 2nd in the global index for the most number of infections after the USA (https://COVID19.who.int/). Over the months, the cases of COVID-19 have been under control and a series of un-lockdowns were observed in India. However, the current statistics show that despite the nation-wide strategic implementation of the vaccination, there is a resurgence of COVID-19 infections, which may result in the various government imposed curbs and lockdowns.

The disease brought along several complications which were novel to the society. Along with the high mortality rates, this pandemic has severe physical, emotional and psychological ramifications. Globally, scientists have invested efforts in diagnosis and treatment of COVID-19. The breadth of the current research on COVID-19 focuses more on its clinical characteristics [2], likelihood of survival [3], genomic characterization of the virus [4] and drug and therapeutic options [5]. Additionally, growing incidences of anxiety, pain, insomnia and distress have been reported widely across nations [6]. Despite the spike in these cases, the myriad psychological impacts of COVID-19 on mental health have not received adequate attention [7]. Similarly, studies in India have been more conceptual in nature and paucity of empirical evidence on assessing the psychological effects and response to COVID-19 has been observed, [8–10].

Psychological distress is defined as a state of emotional suffering typically characterized by symptoms of depression and anxiety, which is recognized as a common mental health problem in the community [11]. In general, mental health and related issues are not recognized in public, and with the global pandemic, these ‘silent’ and insidious issues can go unnoticed. Confinement to physical spaces, lack of mobility, panic buying, fear of contraction, loss of income, adaptation to the new normal and the growing ambiguity were some of the observed collective experiences, affecting the overall well-being during the lockdown [12]. The COVID-19 outbreak has introduced additional stressors that further impinges the mental health status of the general population, making them vulnerable to psychological disorders. Studies have revealed that high distress and anxiety due to COVID-19 has resulted in poor psychological well-being [13], increase in suicidal tendencies [14], exasperated pre-existing mental health conditions [15] to name a few. It has also severely affected the family relationships and social dynamics [16], increase in cases of domestic violence [17], and abuse of alcohol [18].

A rapid increase in the COVID cases and its debilitating impact on the psychological health necessitates an empirical investigation to explore the prevalence and determinants of psychological distress. Such an investigation will help identify the vulnerable groups at risk. This will enable policy makers to design interventions with a targeted approach. Further, the insights of such a study will aid in promoting the protective and minimizing the risk factors. With the impending uncertainty about the end of the pandemic and the emergence of a new strain of the virus, there is a potential for yet another wave, which demands preparedness at the individual and community level. Thus, the objective of the present study is to investigate the psycho-social risk and protective factors to predict distress among general population in India during the COVID-19 pandemic. The findings of the study will contribute in prescribing measures to better manage the psychological crisis and further strengthen mental immunity during a public health emergency.

In the current study, the researchers identified various sources of distress encountered during the ongoing pandemic through interviews with Indian citizens of varied demographics. The interview responses were analysed and various themes were developed that represented different psycho-social stressors (risk and protective factors), which were mapped to the social-ecological model of public health behaviour. This model proposed by Bronfenbrenner (see [19] and references therein), recognizes individuals as embedded within the larger social systems and describes the interactive characteristics of individuals and environments that underlie public health behaviours [20]. This model is popularly used as a conceptual framework for guiding interventions in public mental health [21]. Additionally, this framework provides a holistic perspective to understand health and mental health behaviours.

**Fig 1:**
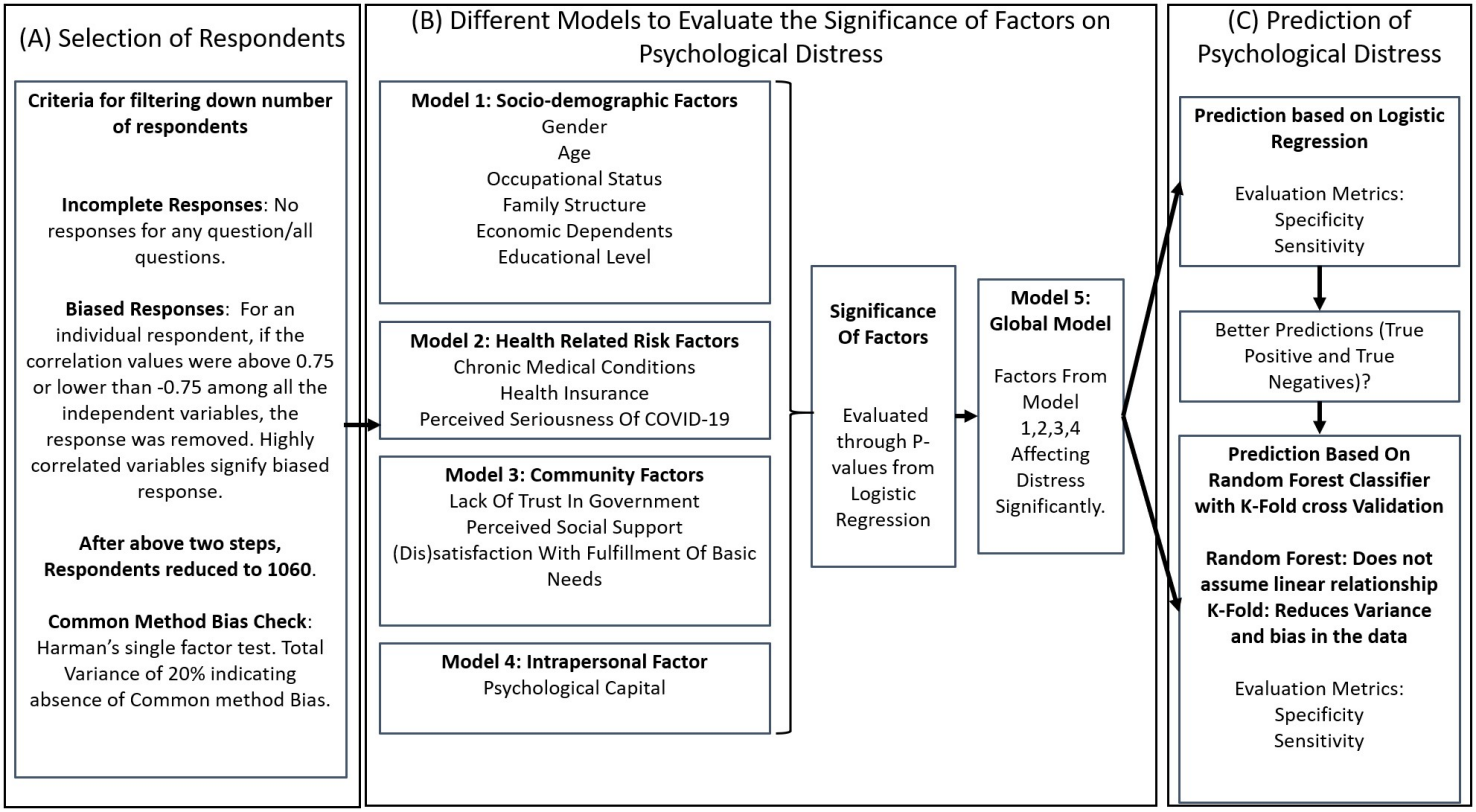
Conceptual framework for the study.

Thus, the present study based on the tenets of the socio-ecological model, organises the psycho-social stressors as individual (socio-demographics and psychological capital), health-related risk (pre-existing medical conditions, health insurance, and perceived seriousness of COVID-19) and community factors (trust in government, social support and fulfillment of basic needs). The conceptual framework of the study is presented in Fig 1.

## 2 Methods

This study adopted a cross-sectional survey approach and a country-wide data was collected. A snowball method of sampling was employed, promoting the study through existing networks and mailing lists. An invitation to participate in an online form was sent through various social media platforms.

### 2.1 Study participants

A total of 1543 respondents from across the country participated in the survey, and designed in the language of English. Among the filled responses, 1060 were considered for the analysis, who largely represented Indian middle to upper socio-economic status (SES)(who could follow English). The study was approved by the Research Review Board, School of Aeronautics, Neemrana, India. Participants were informed about the voluntary participation prior to filing the forms. The participants were informed about the background of involved researchers and about the motive/objective of the survey. The participation was completely voluntary and no intervention of any kind from the researchers conducting the study was involved. The participant was also given an option to skip answering any question(s), if s/he may wish to. The above was done to maintain the most salient ethical values such as beneficence, non-maleficence, trust within the investigator/participant and the privacy of personal information. To control the selection and response bias at respondents level, the survey was floated in various states to get the varied demographics. An easy escape route was provided to the participants as they could exit the survey at their will. To tackle the duplication of the response, the option of not being allowed to fill the survey from the same device more than once, was enabled. Finally, to remove the bias from the responses, a statistical procedure was followed. For an individual respondent, if the correlation values were above 0.75 or lower than -0.75 among all the independent variables, the corresponding response was removed, as highly correlated variables signify biased response [22, 23]. Incomplete forms were determined as the another exclusion criteria. The information regarding the participants email was deleted to preserve anonymity. The demographic profiling of the participants is presented in Table 1.

**Table 1:** Association between variables and distress during the COVID pandemic (*N* = 1060).

	*N*(%)	Distress		*χ*^2^/*t*	*p*	VIF
		Low (%)	High (%)			
		489(Low)	571(High)			
**Gender**				48	< 0.001	2.18
Male	45.94	59.92	33.98			
Female	56.06	39.88	66.02			
**Age (years)**				119.5	< 0.001	9.20
21–35	36.98	32.92	40.98			
36–50	30.94	34.97	27.85			
51–65	18.96	19.84	18.91			
> 65	13.11	12.27	12.26			
**Marital Status**				66.62	< .001	5.75
Married	58.96	56.85	60.95			
Unmarried	41.04	43.15	39.05			
**Family Structure**				135.9	< .001	5.74
Nuclear	60.00	64.83	54.99			
Joint	15.00	11.86	17.86			
Not Applicable	25.00	23.31	27.15			
**Education Level**				64.84	< 0.001	1.93
Below Graduation	50.00	46.83	45.88			
Graduation and Above	50.00	53.17	54.12			
**Occupational Status**				20.65	0.002	2.60
Working on site	0.38	22.90	50.96			
Working from home	0.41	42.94	40.98			
Not Applicable	0.21	34.15	8.06			
**Number of Dependents**				15.72	0.04	2.52
0	21.98	26.99	16.99			
1	40.94	31.90	47.99			
2	20.94	20.86	20.84			
> 2	16.13	19.84	14.19			
**Pre-existing medical conditions**				99.61	< .001	6.76
Unknown	31.04	37.01	25.04			
Yes	45.00	32.92	54.99			
No medical History	23.96	32.11	19.96			
**Have Health Insurance Policy**				9.45	0.014	5.87
Unsure	18.02	25.97	11.03			
Yes	46.04	50.10	42.03			
No	35.94	3.48	46.94			
**Perceived seriousness of COVID-19** (M (SD))	3.68(0.89)	2.84 (0.37)	4.38(0.49)	34.18	< 0.001	6.68
**Lack of Trust in Government** (M (SD))	3.23(1.08)	2.52(0.64)	4.26(0.44)	14.49	< 0.001	3.12
**Social Support** (M (SD))	3.38(1.00)	4.31(0.46)	2.6(0.58)	14.18	< .001	7.86
**Dissatisfaction with fulfillment of basic needs** (M (SD))	3.80(0.90)	2.86(0.38)	4.44(0.50)	2.92	0.004	1.52
**Psychological capital** (M (SD))	3.31(1.10)	4.36(0.39)	2.53(0.59)	16.62	< 0.001	2.04

### 2.2 Measures

#### 2.2.1 Socio-demographic factors

The first part covered the socio-demographic details, which included age, gender, marital status, occupational status, family structure, number of economic dependents, educational level, and the place of residence.

#### 2.2.2 Other factors

The second part included questions anchored to the constructs of the study. Following are the measures used for the present study.

*2.2.2.1 Health-related risk factors*. Pre-existing medical condition was assessed by a single item in which respondents were asked to report if they were suffering from any chronic medical condition. The construct of Health Insurance was also measured by one item in which respondents reported if they possessed a health insurance policy that covered COVID related expenses. The degree of perceived seriousness of COVID-19, was measured by a single item on a 5-point scale (1 = not serious to 5 = extremely serious) which was adapted from the standardized questionnaire on risk perception of an infectious disease outbreak [24].

*2.2.2.2 Community factors*. Trust in Government (lack) was assessed to gauge the level of trust in Government’s capability in managing the crisis. 5 items were used to measure this construct on a 5-point scale (1 = strongly agree to 5 = strongly disagree). 2 items were adapted from Ma et al [25], while the other 3 items were self-developed based on the verbatim interview responses. The degree of social support in terms of availability of others to talk about one’s problems during the crisis was measured by employing 4 items from the appraisal subscale of Interpersonal Support Evaluation List-12 [26]. The items were responded on a 4-point rating scale (1 = definitely false to 4 = definitely true). To measure the (dis)satisfaction with the fulfillment of basic needs, the participants were asked to report the extent to which they were satisfied with the availability and accessibility of basic resources which included water, food, medicines, electricity and internet connection. This was measured on a 5-point scale (1 = unsatisfactory and 5 = extremely satisfactory).

*2.2.2.3 Intrapersonal factors*. Psychological capital defined as an individual’s positive psychological resource was measured through a standardized scale-PCQ-12 [27] that assessed the four integrating dimensions, namely, self-efficacy (3 items), hope (4 items), optimism (2 items) and resilience (3 items) on a 6-point scale (1 = strongly disagree to 6 = strongly agree). A summated score was considered for the study.

*2.2.2.4 Psychological distress*. It was assessed using 6 items Kessler Distress scale (K6) [28]. The items are used for screening major depression and generalised anxiety disorder and asks respondents how frequently they have experienced relevant symptoms during the past month. Each item had five options ranging from 0 (never) to 4 (all of the time).

The instruments designed for this study included items, that were self-developed exclusively for the study, while some were adapted from standardized scales, available and open for academic purposes. Prior permission to use standardized scales was sought by sending an email to the scale developers/ paper authors. The psychometric properties of the instrument were established. For the variables under study, the items were factor analyzed using principal components analysis with promax rotation which yielded an 6-factor structure explaining 67% of the total variance. The Kaiser–Meyer–Olkin (0.883) and Bartlett’s Test of Sphericity (18626, p>0.01) indicated that the factor model is appropriate. Confirmatory Factor Analysis was performed to test the validity measures. Construct validity of the measures was assessed by Factor Loading, Composite Reliability (CR) and Average Variance Extracted (AVE) [29]. All factor loadings were found to be significant and ranged from 0.70–0.82. Reliability results for all constructs were above 0.70, indicating satisfactory reliability [29]. Convergent validity was measured using the average variance extracted (AVE). All constructs exhibited an AVE greater than 0.50, indicating convergent validity. Discriminant validity was confirmed as the square roots of the AVE were larger than the coefficients of the bivariate correlation. Further, to check for the common method bias, Harman’s single-factor test was performed, where all constructs were subjected to Exploratory Factor Analysis with an unrotated factor solution to determine the number of factors necessary to account for the variance. The results revealed a total variance of 20% indicating an absence of common method bias.

### 2.3 Data analysis

The data analysis and modelling were performed using machine learning libraries based on statsmodels in Python. To test the multi-collinearity among considered variables, variance inflation factor (VIF) was calculated. None of the independent variables were dropped as their VIF was found to be less than 10 [30–32]. To explore the relationship of dependent variable (psychological distress) with independent variables, bivariate analyses were performed, including chi-squared test for discrete and independent *t*-test for continuous variables, which have been reported in Table 1. The factors with *p* values equal to or less than 0.05 were considered to be significant. In the bivariate analysis, all the variables had *p* value less than 0.05 and hence no conclusion could be drawn about insignificance of any factor. To further investigate the statistical relevance of the predictors of distress, four logistic regression models were built [33], model 1 with socio-demographic variables, model 2 with health-related risk variables, model 3 with community variables, and model 4 with intrapersonal factor. Finally, a global model (Model 5) was constructed with only variables which had *p* value ≤ 0.05 in each of the individual models. In order to quantify the magnitude of the effect produced by independent variables with respect to the psychological distress, the effect size was measured through their odds Ratios (ORs) with a 95% confidence interval [34, 35]. Accuracy, specificity and sensitivity were used as performance metrics for the models, since accuracy alone is not a good metric to indicate the correctly classified individuals under both low and higher distress [36]. The coefficient of determination, R^2^ value, was considered as a measure of the goodness-of-fit of the model. As the accuracy of the logistic regression classifier on the global model was found to be less than 85%, thus random forest classifier was also employed to increase the overall accuracy of classification. *K*-fold cross validation, with *K* = 5, was employed so that the reported accuracy scores were not higher due to the over-fitting [37, 38].

## 3 Results

### 3.1 Psychological distress

Psychological distress which was initially categorized into a five point scale was re-categorized into two categories, low distress and high distress using median as a cut-off point [39]. Evaluating a cut-off point of 3 for distress, the results showed that 53.86% of the people considered for analysis were under high distress.

### 3.2 Independent variables and distress

Table 1 represents the descriptive statistical analysis of the various independent variables, in relation to the response variable distress.

### 3.3 Prediction of distress

This section details the results of the logistic regression models, which were built across the various predictors of the survey. The results indicate that the global regression model (Model 5) is able to explain the variability of the data to 42.4%. Logistic regression models are displayed in Table 2. All the models had *p*-value less than 0.001.

**Table 2:** Logistic regression models.

Variables		Model 1	Model 2	Model 3	Model 4	Model 5
		OR (95% CI)	OR (95% CI)	OR (95% CI)	OR (95% CI)	OR (95% CI)
		SocioDemographic	Health-Related	Community	Intrapersonal	Global
	Regression Models	R^2^ = 0.355	R^2^ = 0.266	R^2^ = 0.345	R^2^ = 0.0624	R^2^ = 0.424
		(72/84%)	(67/65%)	(85/71%)	(49/57%)	(77.5/86%)
**Sociodemographic**	**Gender (ref. Male)**	2.0023**	NA	NA	NA	1.425**
		(1.507, 2.660)				(1.011, 2.008)
	**Age (in yrs) (ref**. 21–35)					
	36–50	0.410**	NA	NA	NA	0.679*
		(0.289, 0.581)				(0.467, 0.987)
	51–65	1.002	NA	NA	NA	NA
		(0.68, 1.478)				
	> 65	0.945	NA	NA	NA	NA
		(0.541, 1.648)				
	**Marital Status (ref. Not Married)**	1.159	NA	NA	NA	NA
		(3)				
	**Family Structure (ref single)**					
	Nuclear	0.23**	NA	NA	NA	1.311
		(0.151, 0.351)				(0.639, 2.689)
	Joint	2.089**	NA	NA	NA	1.442
		(1.228, 3.554)				(0.828, 2.513)
	**Education Level (ref <Graduation)**	1.137	NA	NA	NA	NA
		(0.776, 1.667)				
	**Occupational Status (ref. Work From Home)**					
	Working on site	2.428**	NA	NA	NA	1.592**
		(1.713, 3.441)				(1.131, 2.241)
	Not Working	1.367	NA	NA	NA	NA
		(0.959, 1.949)				
	**No. of Dependents (ref Zero)**					
	1	2.38**	NA	NA	NA	0.976
		(1.777, 3.187)				(0.646, 1.472)
	2	0.757	NA	NA	NA	NA
		(0.439, 1.305)				
	> 2	0.794	NA	NA	NA	NA
		(0.515, 1.225)				
**Health-Related**	**Pre-existing medical conditions (ref Unknown)**					
	Yes	NA	2.445**	NA	NA	1.682*
			(1.779, 3.360)			(1.056, 2.680)
	No	NA	0.813	NA	NA	1.407
			(0.525, 1.258)			(0.743, 2.664)
	**Health Insurance (ref. Unsure)**					
	Yes	NA	0.791	NA	NA	NA
			(0.579, 1.081)			
	No	NA	3.500**	NA	NA	1.884*
			(1.722, 7.111)			(1.105, 3.214)
	Perceived seriousness of COVID-19	NA	1.104	NA	NA	1.239*
			(0.926, 1.316)			(1.013, 1.515)
**Community**	Lack of Trust in Government	NA	NA	1.497	NA	1.246*
				(1.285, 1.745)		(1.02, 1.521)
	Social Support	NA	NA	0.39**	NA	0.646*
				(0.33, 0.45)		(0.435, 0.961)
	Dissatisfaction with fulfillment of basic needs	NA	NA	3.235**	NA	1.592*
				(2.497, 4.190)		(1.004, 2.524)
**Intrapersonal**	Psychological capital	NA	NA	NA	0.689**	0.467*
					(0.623, 0.761)	(0.312, 0.698)

* *p* < 0.1;

** *p* < 0.05;

NA: Not Applicable

R^2^ = Model explained variance (sensitivity / specificity)

OR(95% CI): Odds Ratio (Confidence Interval at the 95% level).

Model 1 (Sociodemographic variables) had an R^2^ value of 35.5% with *χ*^2^ = 809. It correctly classified 80.1% of respondents with specificity and sensitivity being 72% and 84%, respectively. The participants in the age group of 36–50 years (OR = 0.410, 95% CI = (0.289, 0.581)) and the ones with nuclear family structure (OR = 0.23, 95% CI = (0.151, 0.351)) were less likely to be distressed as compared to the ones in the age group of 21–35 years and the ones living alone respectively. The participants living in joint family structure (OR = 2.089, 95% CI = (1.228, 3.554)), the female participants (OR = 2.0023, 95% CI = (1.507, 2.660)), the ones working on site (OR = 2.428, 95% CI = (1.713, 3.441)) and the ones with only one dependent (OR = 2.380, 95% CI = (1.777, 3.187)) were more likely to be distressed than the ones living alone, males, the ones working from home and the ones with no dependents respectively.

Model 2(Health-related risk variables) had an R^2^ value of 26.63% with *χ*^2^ = 224. The participants with pre-existing medical conditions(OR = 3.066, 95% CI = (2.223, 4.228)) and the ones with no health insurance (OR = 3.352, 95% CI = (2.058, 5.462)) were more likely to be distressed than the ones who were unsure about their medical history, and who were not sure if they had a health insurance for COVID-19, respectively. The model correctly classified 67.5% of respondents with specificity and sensitivity being 67% and 65%, respectively.

Model 3 (Community variables) showed a predictive ability of 34.47% with *χ*^2^ = 73.28, correctly classified 79.3% of respondents with specificity and sensitivity being 85% and 71%, respectively. The participants with lack of trust in government (OR = 1.497, 95% CI = (1.285, 1.745)), and the ones who were dissatisfied with the fulfillment of basic needs (OR = 3.235, 95% CI = (2.497, 4.190)) were more likely to be distressed. Those with high social support (OR = 0.387, 95% CI = (0.333, 0.449)) were less likely to be distressed.

Model 4 (Intrapersonal variable/Psychological Capital) showed a predictive ability of 6.23% with *χ*^2^ and correctly classified 61.1% of respondents with specificity and sensitivity being 49% and 57%, respectively. The participants with psychological capital (OR = 0.689, 95% CI = (0.623, 0.761)) were less likely to be distressed.

Model 5 (Global Model), had a predictive ability of 42.4% and high chi-squared value (*χ*^2^ = 619), which represents good fit to the global model [33]. The significant predictive variables that showed the greater weight, with OR greater than 1, were gender, specifically female (OR = 1.425, 95% CI = (1.011, 2.008)), occupational status as working on site (OR = 1.592, 95% CI = (1.131, 2.241)), awareness about pre-existing medical conditions (OR = 1.682, 95% CI = (1.056, 2.680)), absence of health insurance policy (OR = 1.884, 95% CI = (1.105, 3.214)), high perceived seriousness of COVID-19 (OR = 1.239, 95% CI = (1.013, 1.515)), lack of trust in government (OR = 1.246, 95% CI = (1.020, 1.521)) and dissatisfaction with fulfillment of basic needs (OR = 1.592, 95% CI = (1.004, 2.524)). The predictors with OR less than 1 were psychological capital, social support and age group of 36–50 years. The remaining variables, family structure, number of dependents and absence of pre-existing medical conditions were insignificant.

The final model had a predictive ability of 42.4%, correctly classifying 82.2% of respondents with specificity and sensitivity being 77.5% and 86% respectively when modelled through logistic regression. In order to increase classification performance, random forest classifier was employed on the predictors of the global model (Model 5). The accuracy of this model was 92%, with specificity and sensitivity of 94.5% and 88.3% respectively. Even though, the correct classification of people under high distress increased by 2.3%, in comparison to logistic regression, the accuracy of the random forest classifier was 17.1% higher for people with low distress.

## 4 Discussion

The present study aimed at identifying the psycho-social factors predicting psychological distress, experienced amongst the general population in India, in the times of COVID-19 pandemic. As mentioned in §3.1, prevalence of distress was observed. The results of the study indicate that the socio-demographic, health-related risk, community and intrapersonal factors have a significant influence on distress.

Within the socio-demographic factors, the respondents in the age group of 21–35 years were found to be more prone to distress (40.98%) as compared to the other age groups considered for the study. These findings are consistent with those from previous studies during an epidemic [40–42], which showed that younger population was associated with an increased risk of distress. The studies have also shown that older adults have increased resilience to psychopathologies such as post-traumatic stress disorder after an emergency due to natural disasters, indicating that older population are better equipped to handle stressful situations. [43–46]. Studies suggest that younger population experiences higher anxiety due to proximity to contamination, information overload through social media and a poor tolerance of uncertainty, which might explain the findings of the present study [47]. This study reported that the females experienced higher distress (66.02%) than their counterparts, which is in line with the previously available extensive epidemiological literature. In accordance with other studies carried out in China during COVID-19 pandemic [7, 48, 49], women and young adults were the ones that suffered the greater psychological impact. Generally, women are the informal caregivers within families, and with the additional restrictive measures (such as closure of the educational institutions and care for elderly), might have led to an increased burden at home, thereby explaining higher distress [50]. Increased domestic responsibilities could further impact their work performance, whether working onsite or remotely, compounding their levels of distress [51]. Yet another reason could be increased occurrences of domestic violence against women that were recorded during times of crisis and quarantines [40, 51, 52]. This study found that the respondents who were working on site were more distressed (50.96%) than those working from home or not working. Similar results were found in a research amongst general adult population in Spain [53]. Perceived inadequate workplace protective equipment supply, fear of contracting the disease and the stigma associated with it, during COVID-19 pandemic significantly led to employee stress which explains why employees working on site were highly likely to be distressed [54]. Hence, fearing the consequences, employees preferred working from home during the COVID-19 pandemic.

Amongst the health-related risk factors evaluated in this study, all three sub-factors were found to be significantly contributing to distress. Extensive review of literature suggests that the following sub-factor, is rarely explored in the epidemiological literature. The findings revealed that people who had no health insurance were more stressed (46.94%) than the ones who were not sure if their insurance covered COVID-19 related expenses. The limited available literature showed that having difficulties in covering monthly expenses in non-pandemic times was positively correlated with distress, and it is of no surprise that the crisis situation could aggravate the stress levels [55]. Those with a history of pre-existing medical conditions reported higher distress (54.99%) than the ones who were not sure if they ever suffered from one. Similar findings suggest that poor perception of physical health [56] and a history of pre-existing medical conditions or illnesses [7] can lead to higher stress.

Previous literature has also asserted that perceived risk of contracting the disease is a significant stressor. The results of this study further validate the past findings as the respondents who perceived COVID-19 more seriously reported higher levels of distress(M = 4.38, SD = 0.49). An Asian study [57] further highlighted and confirmed the significant role of this factor in explaining poor mental health during COVID-19, in addition to other factors such as misinformation and social isolation in contributing to stress and mental morbidity. Perhaps, lack of authentic information, ambiguity about the novel Coronoavirus along with less confidence in healthcare fraternity to treat COVID-19 could explain higher stress [58, 59].

The community factors assessed in the present paper showed higher association with the experience of distress. The first sub-factor i.e. lack of trust in the government initiatives to combat COVID-19 positively predicted high levels of distress(M = 4.26, SD = 0.44), much in line with the recent studies that suggest public’s trust in the government is critical for community mental health. Infact, [60] reports that the lack of trust can also hamper emergency and recovery procedures, harming general public during any given crisis situation, like COVID-19 pandemic. The resulting unrest and chaos, may probably, lead to an increase in distress. The second sub-factor, social support is well established as a protective factor, buffering the harmful effects of distress. Previous studies have highlighted the role of social support in reducing anxiety and stress, [61], with which the results of this study resonate (M = 4.31, SD = 0.46). The final sub-factor, the (dis)satisfaction with fulfillment of basic needs during the pandemic, which included food, water, electricity, medicine and internet, was found to have a positive significant influence on distress (M = 4.44, SD = 0.50). A paucity of research concerning availability or lack thereof in determining distress was observed. Limited research findings exhibited that availability of local medical facilities influenced levels of distress among Chinese population [62]. In fact, the basic underlying assumptions of need theories also state that only when human needs are fulfilled sufficiently, people experience happiness [63]. Due to stringent lockdown measures, panic buying due to perceived shortage of basic resources was reported, which could accentuate the stress levels [8].

The final factor for the study was psychological capital(PsyCap) and it was found that the respondents with higher PsyCap were less likely to be distressed(M = 2.53, SD = 0.59). This factor has been explored to a very limited extent during a pandemic or crisis, in the literature, which reflects a very pertinent gap in terms of research in intrapersonal factors. A meta-analytic study [64] on PsyCap states that those low in PsyCap are more prone to stress. The review further suggested that PsyCap’s agentic thinking has a motivating impact that can enhance internalization, determination, and pathways thinking, which contradict with the ‘giving up’ and despair associated with cynicism, thereby protecting an individual, from the toxic effects of stress. The extant literature [65] has established that the more positive capacities one uses, the ability to deal with stressful situations improves considerably.

## 5 Conclusion

This study confirms the prevalence of distress experienced by the citizens of India during the pandemic and provides pragmatic implications for stress management at macro and microlevels during an epidemiological crisis. Based on the findings it is suggested that public health machinery must conduct mental health audits during epidemiological emergencies, which are critical for effective management of community mental health. This can further aid in delivering targeted psycho-social interventions for the identified vulnerable groups (which includes females, 21–35 years old citizens, the ones with pre-existing medical conditions, and those working on site). Several initiatives are currently being undertaken by the Government such as providing tollfree helplines and tele counselling services for mental health assistance during the COVID-19 pandemic. Similarly, resource materials and manuals on managing stress during COVID, yoga and meditation, etc. are available to the public on the MOHFW-GOI website [66]. However, for effective reachability of such interventions, the services and facilities need to be curated to the specific needs of the identified susceptible groups.

Since the study revealed that those who did not possess an insurance policy for treatment of COVID-19 are more prone to distress, thus, it is recommended that the Government regulates and mandates the insurance companies to provide diagnostic and treatment coverage for the disease in the form of protective care. This will help to deal with any medical emergency with hopes of leading a worry-free life ahead. Further, the insurance companies must make efforts to communicate the coverage in their basic plans.

As public’s lack of trust towards the Government policies and initiatives around COVID-19 was identified as a significant predictor of distress, thus a constant attempt by the public authorities to understand the community’s perception of their policies must be undertaken. This will aid in employing effective strategies to inform, educate and communicate the public about such initiatives, thereby fostering trust in the Government. People’s perception of the seriousness of the disease which was also identified as major source of distress, is highly influenced by Government and social media communication. In this regard, greater caution needs to be exercised while devising risk communication strategies which can include preparedness, response, and recovery phases of a serious public health crisis, rather than just reporting on the number of infected, recovered and death cases. Dissatisfaction with fulfillment of basic needs was found to contribute to distress. Therefore, it is proposed that local authorities invest in assessing the basic needs and reallocating the distribution of basic resources during the crisis.

Human beings are gregarious in nature and we always need social connection in our lives. As reported in the study, social support during crisis, is paramount to cope with stressful situations. Thus, public health measures may focus on promoting a supportive environment: and provide resources to cultivate and maintain a sense of community belongingness. It is well known that external resources are not in our control, and thus it becomes imperative to focus on building and strengthening our internal resources to tide through these times. As the present study recognized positive capacities of resilience, hope, optimism and self-efficacy that constitute PsyCap as a protective factor during stressful situations, hence the Government, and systemic organizations/institutes such as schools, organizations and non-governmental organizations should invest in development of positive capacities for sustainable well-being in the community.

## 6 Limitations

Although the study contributed to the literature of mental health during COVID-19, it also has certain limitations. Since the sample comprised of respondents from restrictive socio-economic status, limited geographical coverage and poor representation from red-zone cities, the results cannot be generalized. The non-probability sampling method(snowball sampling) employed for the study may have potentially affected the generalizability and sample representativeness. As the study followed a cross-sectional approach, it could not capture the changing trends during multiple lockdowns observed in India. Hence, future studies can follow a longitudinal approach to identify and understand the changes which could have occurred over a period of time. Interpersonal factors like family dynamics and relationship with others, could be potential factors influencing distress which can be analyzed in future studies.

## Supporting information

S1 Data (TXT)
